# Probiotic bacteria prevent *Salmonella* – induced suppression of lymphoproliferation in mice by an immunomodulatory mechanism

**DOI:** 10.1186/s12866-017-0990-x

**Published:** 2017-03-29

**Authors:** R. Doug Wagner, Shemedia J. Johnson

**Affiliations:** Microbiology Division, National Center for Toxicological Research, U.S. Food and Drug Administration, 3900 NCTR Rd, Jefferson, AR 72079 USA

**Keywords:** Probiotic, Gene expression, Signal transduction genes, *Salmonella*, Apoptosis

## Abstract

**Background:**

*Salmonella enterica* infections often exhibit a form of immune evasion. We previously observed that probiotic bacteria could prevent inhibition of lymphoproliferation and apoptosis responses of T cells associated with *S. enterica* infections in orally challenged mice.

**Results:**

In this study, changes in expression of genes related to lymphocyte activation in mucosa-associated lymphoid tissues (MALT) of mice orally infected with *S. enterica* with and without treatment with probiotic bacteria were evaluated. Probiotic bacteria increased expression of mRNA for clusters of differentiation antigen 2 (*Cd2*), protein tyrosine phosphatase receptor type C (*Ptprc*), and Toll-like receptor 6 (*Tlr6*) genes related to T and B cell activation in mouse intestinal tissue. The probiotic bacteria were also associated with reduced mRNA expression of a group of genes (*RelB, Myd88, I*κκ*a, Jun, Irak2*) related to nuclear factor of kappa light chains enhancer in B cells (NF-κB) signal transduction pathway-regulated cytokine responses. Probiotic bacteria were also associated with reduced mRNA expression of apoptotic genes (*Casp2, Casp12, Dad1, Akt1, Bad*) that suggest high avidity lymphocyte sparing. Reduced CD2 immunostaining in mesenteric lymph nodes (MLN) was suggestive of reduced lymphocyte activation in probiotic-treated mice. Reduced immunostaining of TLR6 in MALT of probiotic-treated, *S. enterica*-infected mice suggests that diminished innate immune sensitivity to *S. enterica* antigens is associated with preventing lymphocyte deletion.

**Conclusions:**

The results of this study are consistent with prevention of *S. enterica*-induced deletion of lymphocytes by the influence of probiotic bacteria in mucosal lymphoid tissues of mice.

## Background

*Salmonella enterica* is known to evade host defenses and induce persistent infections [1]. *Salmonella* sp. persistence is also aided by a virulence mechanism that causes clonal deletion of high-avidity CD4^+^ T cells [2]. Furthermore, *Salmonella* sp. induces an inflammatory response and secretes a factor that blocks macrophage and dendritic cell migration [3]. The intestinal microbiota can modulate the pathogenesis and host evasion mechanisms of *S. enterica*; for example, commensal *E. coli* improved the host response to *Salmonella* sp. infection by an immune system regulatory effect [4]. The mechanisms of mucosal immune system regulation are only partially understood and the roles of microbes in this regulation are even less well understood. Addition of probiotic bacteria to the indigenous intestinal microbiota also affects mucosal immune regulation.

In a previous study, we observed that bacteria from a commercial probiotic product modified the host immune response to *S. enterica* by enhancing the proliferative response of spleen lymphocytes to *S. enterica* antigens. We also observed that the T and B cell enriched splenocyte fractions had reduced activities of apoptosis-related cysteine peptidase or caspase (CASP) 3 and CASP7 [5]. Caspases are involved in the signaling pathway that directs programmed cell death (apoptosis) in activated immune cells. These results were observed in mice that were not previously immunized to *S. enterica*, suggesting that the innate pathways of immune activation were involved. T cells that have been activated, but lacking secondary signals, activate an intrinsic caspase pathway that is arrested by signals from B- cell CLL/lymphoma 2 (BCL) 2 and its related proteins. The probiotic bacteria may be providing analogs to these secondary signals that maintain the survival of activated lymphocytes.

The present study sought to determine whether the secondary signals are mediated by intermediary antigen-presenting cells (APC), such as dendritic cells and intestinal epithelial cells or by direct contact to lymphocytes. APC would signal to lymphocytes with cytokines to affect a change in their activation and apoptotic status. Expression of these and other signaling molecules in mucosal tissues of probiotic-treated and untreated *S. enterica*-infected mice were compared. A direct T cell response to microbial antigens would have to occur by a yet unrevealed mechanism or possibly by the same pathways that function in the APC. Intestinal epithelial cells are also involved in T cell activation by production of pro-inflammatory cytokines in the presence of commensal or probiotic bacteria [6].

## Methods

### Microorganisms

A blend of bacteria derived from a commercial probiotic product, which contained: *Lactobacillus reuteri, Lactobacillus rhamnosus, Lactobacillus acidophilus, Lactobacillus casei, Lactobacillus gasseri, Bifidobacterium thermophilus, Bifidobacterium longum,* and *Bifidobacterium adolescentis* was used, as previously described [5]. These isolates are available from the corresponding author upon reasonable request. The products were cultured for isolation of component bacteria on de Man Rogosa Sharpe (MRS) agar (REMEL Laboratories) and *Bifidobacterium* agars (Anaerobe Systems) incubated anaerobically for 48 h at 37 °C. Bacterial isolates were identified by their cellular fatty acid methyl ester compositions (Microbial ID, Inc.) and by their 16S rRNA sequences with the MicroSeq 16S rRNA sequence assays (Applied Biosystems, Inc.). Rapid identifications were made with the Biolog Microbial Identification System. Serial dilution plate counts on MRS or *Bifidobacterium* agar were used to quantify the components of the probiotic products. Probiotic products were suspended in drinking water at a concentration of 5 × 10^6^ CFU/mouse and administered to mice with a feeding tube, as previously described [5]. *Salmonella enterica* Serotype Cubana, originally isolated from poultry [5] was grown on Trypticase Soy agar with 5% sheep blood (REMEL) or in Trypticase Soy broth at 37 °C in an atmosphere of 5% CO_2_ and air. The *Salmonella* isolate was originally characterized as Serotype Typhimurium and subsequently determined to be serotype G2 (serovar Cubana) by the Arkansas Regional Laboratory of the Food and Drug Administration (FDA), Jefferson, AR. The isolate is available upon reasonable request from the corresponding author.

### Mice

A total of 4 male and 4 female 8 week-old human microbiota-associated [5] and 4 male and 4 female 8 week-old defined-microbiota BALB/c mice (Charles River) were used in this study with the approval of the Institutional Animal Care and Use Committee of the National Center for Toxicological Research. Sterile water and NIH-31 mouse chow (Purina) were supplied ad libitum to the mice. The probiotic bacteria were administered with a feeding tube to mice 7 days before oral challenge with *S. enterica*. Isolator sterility was assessed with weekly swab cultures on Trypticase soy blood agar plates (REMEL). The cultures were incubated at 37 °C in 95% air, 5% CO_2_ atmosphere overnight and the plates were assessed for bacterial growth. The mice were fed a NIH-31 diet sterilized in the transfer box as previously described [5].

### Experimental design

Control mice were human microbiota-associated in our laboratory, as previously described [5] or specific pathogen free from the vendor and fecal samples were cultured to assure their status. Treatment mice were colonized with the probiotic bacteria blend and fecal samples were collected from the mice 1 day later to culture for the presence of the probiotic bacteria. At 7 days after probiotic treatment, 4 probiotic-treated mice and 4 untreated mice were each orally inoculated with 2 × 10^8^ colony forming units (CFU) of *Salmonella enterica* using a feeding tube. Seven days later, the mice were euthanized and MALT consisting of intestinal lamina propria, Peyer’s patches, and mesenteric lymph nodes were excised for analysis. Spleen cells were isolated and incubated with *S. enterica* antigens and mitogens to evaluate lymphoproliferative effects, as previously described [5]. Total RNA samples were extracted from the tissues and cDNA were generated, from which quantitative real-time-polymerase chain reaction (qRT-PCR) analyses were run to quantify mRNA expressed from genes of the mucosal immune system in probiotic-treated and untreated mice. The data were analyzed to identify cell type markers and intracellular signaling molecules involved in the host response to *S. enterica* that were affected by the probiotic bacteria. Control mice and probiotic-bacteria-treated BALB/c mice were orally challenged with *S. enterica* and pathway-focused gene expression profiles were generated from qRT-PCR expression arrays, as described below, to compare signal transduction in MALT from defined-microbiota mice treated with or without probiotic bacteria and orally challenged with *S. enterica*.

The experiment was repeated on a group of 8 specific pathogen free mice to obtain intestinal lamina propria, Peyer’s patches, and mesenteric lymph nodes for immunolocalization by an immunohistochemical method using antibodies from Santa Cruz Biotechnology. Immunolocalization of CD21^+^ B cells and dendritic cells, CD2^+^ T lymphocytes, and detection of PTPRC, TLR6, and v-rel avian reticuloendotheliosis viral oncogene homolog B (RELB) cellular expression was used to evaluate the probiotic effects that were observed on mRNA expression.

### Antigen preparations

Antigens were prepared from crude lysates of *S. enterica* for in vitro activation and apoptosis assays of lymphocytes collected from the spleens of mice from the experiments, as previously described [5]. Briefly, the entire volume of a 500 ml log phase broth culture of bacteria was centrifuged at 2000 × *g* for 15 min. The bacterial pellet was washed three times with an equal volume of PBS and centrifuged again. The final bacterial pellet was suspended in 10 ml of PBS and passed through a French pressure cell (SLM/AMINCO) at 15,000 lb./in^2^ to disrupt the bacteria. The disrupted bacteria were centrifuged at 2000 × *g* and the protein content of the supernatant was determined by the bicinchoninic acid protein assay (Pierce Chemical Co.) to express antigen mass as mg protein, and used as the antigens for lymphocyte proliferation assays.

### Lymphocyte proliferation assay

Lymphocytes from the spleens of mice treated with probiotics after *S. enterica* challenge were assayed for proliferative responses to *S. enterica* antigens, as previously described [5]. Lymphocyte proliferation assays were performed with the CellTiter Aqueous 96 assay (Promega, Corp.). Lymphocytes from the spleens of experimentally treated mice were prepared and incubated at a density of 5 × 10^5^ cells/well of a 96-well culture plate in RPMI medium (Thermo Fisher Scientific, Inc.) containing *S. enterica* antigens. Antigens were added to 3 wells with spleen cells at a concentration of 10 μg whole cell lysate protein antigen preparation per well. Antigens were incubated with the cells 56 h at 37 °C in a humidified 5% CO_2_ incubator before testing for lymphocyte proliferation. The formation of proliferating clonal clusters was also verified microscopically. The proliferation of lymphocytes in response to the antigens was measured as absorbance of reduced 3-(4,5-dimethylthiazol-2-yl)-5-(3-carboxymethoxyphenyl)-2-(4-sulfonyl)-2H–tetrazolium, inner salt (MTS) at 490 nm, which was measured with a plate reader (Applied Biosystems). The average of three wells per sample was used to determine the mean ± standard error of the mean (SEM) Abs_490_ for three mice per group. Proliferative responses of lymphocytes to antigens were compared as % increases in MTS absorbance as a result of the effects of probiotics.

### Apoptosis assay

Lymphocytes from the spleens of the mice 7 days after probiotic treatment and *S. enterica* challenge were analyzed for activation of caspases 3 and 7 (Apo-ONE, Promega Corp.). Each assay well of a Nunc F16 black Maxisorp 96 well fluorescent assay plate (Thermo Fisher Scientific, Inc.) contained 50 μl of cell suspension in Roswell Park Memorial Institute (RPMI) 1640 medium at a cell concentration of 2 × 10^5^ cells/ml to which was added 10 μg antigen preparation per well. After 56 h incubation at 37 °C in a humidified 5% CO_2_ incubator, 100 μl of working substrate solution was added to each well. The plate was rotated at 300 rpm for 30 min at room temperature. Fluorescence intensities were measured in a plate reader (Applied Biosystems) set with filters for an excitation wavelength of 485 nm and an emission wavelength of 530 nm at 30 min intervals until the rates of increase reached a plateau state. Endpoint fluorescence intensities from each treatment group were compared as the % change in relative fluorescence intensities resulting from probiotic inhibition of CASP3/7activation.

### qRT-PCR array profiling of signaling pathway and cytokine genes

RT^2^ Profiler™ PCR arrays from Qiagen Bioscience were used to assess expression of mRNA for 327 genes involved in the host response to bacteria in the mucosal immune tissues of the mouse GI tract. Arrays for genes involved in apoptosis (PAMM-012 Mouse Apoptosis Array), for genes involved in NF-κB activation (PAMM-025 Mouse NF-κB Array), and for mouse T and B cell activation markers (PAMM-053) were used according to the manufacturer’s instructions. Total cellular RNA from Peyer’s patches, mesenteric lymph nodes, and lamina propria from 8 mice were isolated using ArrayGrade total RNA isolation kits (Qiagen, Inc.). The RNA samples were treated with DNAse-1 (Thermo Fisher Scientific, Inc.) reverse-transcribed with the RT^2^ PCR Array first strand kit and the resulting cDNA was analyzed by real-time PCR for detection in a BioRad Pci,Q5 instrument. The housekeeping genes used in the study were: beta glucuronidase*,* hypoxanthine phosphoribosyltransferase 1*,* heat shock protein 90 alpha family class B member 1*,* glyceraldehyde-3-phophate dehydrogenase*,* and beta actin. Results of the PCR array experiment were analyzed with the Excel™ (Microsoft Corp.) template provided by Qiagen, Inc. to determine the key signal transduction pathways and immune system cells involved in the probiotic effects.

### Immunolocalization of responses to probiotic bacteria in the murine MALT

The gene expression profiling was used to indicate specific mouse immune system genes that respond to the effect of probiotic bacteria in response to *S. enterica*. Two-color immunohistochemistry was used to determine the cell types involved with specific gene product markers and their tissue locations. Toxicologic Pathology Associates (Jefferson, Arkansas) prepared frozen sections of the MALT tissues from 8 mice and conducted the immunolocalization on those sections for cell markers and specific intracellular signal transduction gene products. Antibodies labelled with horseradish peroxidase specific for CD21, CD2, PTPRC, TLR6, and RELB were purchased from Santa Cruz Biologics. Densitometry of the stained areas in Peyer’s patches, lamina propria of intestinal villi, or the cortical and paracortical regions of mesenteric lymph nodes were measured by the Positive Pixel Count Algorithm from Aperio Technologies (Leica Biosystems).

### Analysis of data

Evaluation of statistically significant differences between the results from treatment groups and control groups were determined with Repeated Measures Analysis of Variance and Bonferroni’s post tests using Prism v.6.0 software (GraphPad Software). Numerical count data were log_10_ transformed prior to statistical analysis to make the data better fit a normal distribution. Statistical significance was defined at *P* < 0.05.

## Results

### Persistence of probiotic bacteria and *Salmonella* sp. in the murine GI tracts

In our previous study [5], colonization of germfree mice by probiotic bacteria was easily verified by microbial culture from feces, but the mice in this study had conventional microbiota. The presence of *L. reuteri*, which is not a component of the BALB/c mouse colony microbiota (Charles River) in the defined-microbiota mice in the present study was confirmed by culture on MRS agar and microbial identification of colonies from feces at 7 days after oral feeding of 8.0 log_10_ CFU of the probiotic bacterial mixture. Infection of mice was confirmed by recovery of *S. enterica* from the mice 7 days after oral challenge (Table 1).

**Table 1:** Numbers of bacteria recovered from experimental mice

Animal^a^	Probiotic-treated	Salmonella detected (CFU/mL)^b^	No. total lactobacilli (CFU/g)^c^
1	-	2.2 × 10^6^	5.0 × 10^10^
2	-	6.7 × 10^6^	3.3 × 10^10^
3	-	1.6 × 10^7^	4.8 × 10^10^
4	-	2.8 × 10^6^	4.2 × 10^10^
5	+	0	6.9 × 10^11^
6	+	0	4.2 × 10^11^
7	+	0	4.4 × 10^11^
8	+	7.6 × 10^6^	5.2 × 10^11^

^a^Mice 1–4 were non-treated with probiotic lactic acid bacteria. Mice 5–8 were orally challenged with 1 × 10^8^ CFU probiotic bacteria mixture. Seven days later, all mice were orally challenged with 1 × 10^8^ CFU/mL *S. enterica*. ^b^The number of *S. enterica* isolated from feces of mice 7 days after challenge were enumerated on SS agar plates. ^c^The numbers of lactic acid bacteria were enumerated on MRS agar plates and reported as CFU/g feces 7 days after *S. enterica* challenge

*S. enterica* was recovered from one of the probiotic-fed mice, and all of the control mice 7 days after oral *Salmonella* challenge. Table 1 also shows numbers of lactic acid bacteria that were recovered from mice 7 days after oral *S. enterica* challenge and grown on MRS agar.

### Probiotic bacteria prevented immunosuppression of T cells in mice to *S. enterica* antigens

**Fig. 1:**
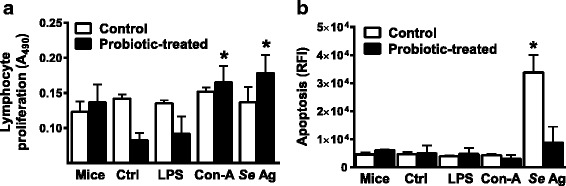
Lymphocyte proliferation and apoptosis responses. Proliferation of splenocytes from uninfected mice (Mice), *S. enterica*-infected BALB/c mice (Ctrl), or *S. enterica*-infected, probiotic-treated mice to LPS (LPS), concanavalin-A (Con-A), or soluble *S. enterica* antigens (*Se* Ag) was measured as change in absorbance of MTS at 490 nm (**a**). Apoptosis was measured as activation of caspases 3 and 7 in splenocytes from uninfected mice (Mice), *S. enterica*-infected mice (Ctrl), or *S. enterica*-infected, probiotic-treated mice to LPS (LPS), concanavalin-A (Con-A), or soluble *S. enterica* antigens (*Se* Ag), detected as relative fluorescence intensity (RFI) of caspase substrate, were compared (**b**). The *asterisks* indicate statistically significant differences between control (Ctrl) and treated groups by ANOVA, *P* < 0.05

Lymphocytes from the spleens of BALB/c mice challenged with *S. enterica* only for 7 days did not proliferate in response to the B-cell mitogen lipopolysaccharide (LPS) or to the T cell mitogen concanavalin-A, nor did they respond to soluble antigens from *S. enterica* (Fig. 1a). This lack of responsiveness was observed under similar conditions in our previous report [5]. Decreased lymphoproliferation of splenocytes in mice treated with probiotics was observed in the control treatment and LPS treatment groups. Splenocytes from mice that were fed the mixture of probiotic bacteria prior to *S. enterica* challenge had significant proliferative responses to concanavalin-A and the *S. enterica* antigens (Fig. 1a), showing a preventative effect of the probiotic bacteria on *S. enterica*-induced immunosuppression. The lymphoproliferative responses to *S. enterica* LPS were not significantly changed by probiotic bacteria compared with the *S. enterica*-treated control mice.

The probiotic bacteria altered the activation of cellular apoptosis in lymphocytes, as reported previously [5]. In the present study, activation of CASP3 and CASP7, which mediate lymphocyte apoptosis, was significantly increased in *S. enterica*-infected BALB/c mice, compared to the response to LPS or concanavalin-A, but was strongly induced by *S. enterica* antigens (Fig. 1b). The mice that were fed probiotic bacteria before *S. enterica* challenge did not have a significant increase in lymphocyte CASP3 and CASP7 activation by *S. enterica* antigens (Fig. 1b), which suggests that strong *S. enterica* induction of T cell apoptotic responses was suppressed by the probiotics.

### Probiotic bacteria altered mRNA expression of genes involved in responses to *S. enterica* infection

The presence of probiotic bacteria induced changes in expression of immune response genes and cellular locations in the MALT of mice infected with *S. enterica*. There was a general increase in T cell function and a decrease in inflammatory responses, as evidenced by increased expression of T cell activation genes and apoptosis-related genes and decreased gene expression of NF-κB signal transduction pathway components and proinflammatory cytokines, respectively (Table 2). Probiotic lactobacilli had significant effects on the expression of mRNA from genes involved in B and T cell activation, signal transduction by the NF-κB pathway, and apoptosis, when the mice were challenged with *S. enterica*. Expression of the mRNA for the lymphokines interleukin (IL) -12 p40 (*Il12p40*) and *Il4* were reduced, while *Il10* mRNA was significantly increased by the presence of probiotic lactobacilli (Table 2). Other genes involved in lymphocyte activation were also affected by probiotic bacteria. Nearly 3-fold induction of mRNA for *Cd2*, and *Ptprc* genes (Table 2) indicates activation and differentiation of B and T cells. Other components of the T and B cell activation signal-transduction pathways were also induced (Table 2). Since activated splenocytes proliferated in response to concanavalin-A, but not *Salmonella* LPS (Fig. 1a), it appears that mostly T cell activation accounts for the increased expression of the lymphocyte activation genes listed in Table 2. This conclusion is supported by the result that expression of the mRNA for *Tlr*4, the protein of which is a receptor for *S. enterica* lipopolysaccharides on APC, was not affected by probiotic bacteria (Table 2). Decreased expression of *Tlr*1 and increased expression of *Tlr6* in MALT suggests that probiotic bacteria modified the spectrum of bacterial lipoproteins that could be detected by APC in the MALT. RNA for *Cd21* was not detected in the *S. enterica* challenged mice that were not treated with probiotic bacteria. It was present in samples of MALT from the probiotic-treated mice, consistent with the possibility that probiotics prevented the loss of antigen-specific lymphocytes at sites of infection.

**Table 2:** Gene Expression Effects of Probiotic Bacteria on *S. enterica-*Challenged Mice (Fold Change in mRNA Expression vs. Control)

Gene-coded protein/Function	Fold Change^a^
B and T cell activation genes^b^	
*Cd2*/Induces IFN-γ, interacts with PTPRC	+ 2.9
*Cd21* (*Cr2*)/B cell complement receptor	+ 3.0
*Hells*/Helicase - Cell growth, DNA methylation	+ 2.5
*Hsp90aa1*/AKT signal transduction pathway, protein chaperon	+ 2.3
*Il4*/TH-2 cytokine (IL-4)	- 2.6
*Il10*/TH-2 cytokine (IL-10)	+ 3.6
*Il12b*/TH-1 cytokine (IL-12p40)	- 2.7
*Impdh*/Cell growth	+ 2.5
*Nkx2.3*/Lymphocyte cellular differentiation	+ 2.3
*Prkcd*/B-cell signal transduction and apoptosis	+ 2.2
*Prlr*/Prolactin receptor	+ 2.1
*Ptprc*/(CD45/B220) cell growth/differentiation	+ 3.0
*Tlr1*/Proinflammatory receptor for microbes	- 2.5
*Tlr6*/Proinflammatory receptor for microbes	+ 2.3
*Vav1*/T and B cell activation via JNK and P38	+ 2.1
*Wwp1*/E3 ubiquitin protein ligase	+ 2.4
*Zap70*/T cell receptor cofactor	- 2.4
*Nf-κb* signal transduction genes^c^	
*Bcl2*/Anti-apoptotic signaling	- 1.1
*Bcl2l10*/Pro-apoptotic, pro-inflammatory signaling	- 1.8
*Cflar*/Pro-apoptotic signaling	- 1.2
*Egr1*/B-cell receptor signaling	- 2.1
*Fasl*/Intercellular signaling, pro-apoptotic	- 3.3
*Fos*/Intracellular signaling, pro-apoptotic	- 3.1
*Icam1*/Leukocyte movement, signaling	- 2.3
*Iκκa* (*Chuk*)/Pro-inflammatory activator of NF-κB	- 2.1
*Irak2*/Induces NFκB nuclear translocation	+ 2.3
*Jun*/(AP-1) B-cell and T-cell receptor signaling	+ 2.1
*Mekk*/(*Map3k1*) Pro-inflammatory	- 2.2
*Myd88*/Pro-apoptotic and inflammatory adapter	- 3.8
*Nfkbia*/Blocks NFκB nuclear translocation	- 2.1
*Relb*/NFκB cofactor activation in B-cells	- 3.0
*Ripk1*/Pro-inflammatory, apoptosis regulatory	- 1.1
*Rnf7*/IκB ubiquitination – pro-apoptotic, pro-inflammatory	- 3.8
*Traf2*/Pro-inflammatory, apoptosis regulatory	- 1.7
Apoptosis genes^d^	
*Akt1*/Anti-apoptotic	- 2.1
*Bad*/Anti- apoptotic	- 2.2
*Card10*/Proinflammatory via NFκB, pro-apoptotic	- 4.3
*Casp2*/Pro-apoptotic	- 2.3
*Casp12*/Pro-apoptotic	- 2.8
*Dad1*/Possible apoptosis inhibitor	- 3.1
*Fadd*/Pro-apoptotic	- 2.2
*Pak7*/Protein kinase induces proliferation/ anti-apoptotic	- 4.2
*Tnf*/Pro-apoptotic cytokine	- 2.4

^a^The mean (*n* = 4/group) fold change in qRT-PCR threshold cycles (C_T_) between tissue RNA samples amplified from probiotic-treated versus untreated control mice were calculated as 2^(−ΔΔCT)^, where ΔC_T_ is the housekeeping gene-normalized average C_T_ and ΔΔC_T_ is the ΔC_T(treatment)_- ΔC_T(control)_. Positive values indicate increased expression and negative values indicate reduced expression

^b^Results from Qiagen PAMM-053 Mouse T and B Cell Activation Array

^c^Results from Qiagen PAMM-025 Mouse NF-κB Array

^d^Results from Qiagen PAMM-012 Mouse Apoptosis Array

Additional effects of the probiotic lactobacilli were observed in genes that relate to the NF-κB signal transduction pathway that controls expression of cytokine genes in response to receptor detection of microbial antigens. mRNA expression of several signaling protein genes CASP8 and FADD like apoptosis regulator (*Cflar*), *Casp8*, receptor TNFRSF-interacting serine threonine kinase 1 (*Ripk1*), TNF receptor associated factor 2 (*Traf2*), *Bcl10*, and *Bcl2*) was slightly reduced, but not to the 2-fold change level of significance (Table 2). Significant reduction (*P* < 0.05) in mRNA expression of *RelB*, myeloid differentiation primary response gene 88 (*Myd88*), ring finger protein 7 (*Rnf7*), inhibitor of light polypeptide gene enhancer in B cell kinase alpha (*Iκκα*), and Map/Erk kinase kinase (*Mekk*) are indicative of a response that suppresses transduction of proinflammatory signals in lymphocytes (Table 2). The increased mRNA expression of interleukin-*1 receptor-associated kinase* 2 (*Irak2*) and v-jun sarcoma virus 17 oncogene homolog (*Jun*) induced by probiotic bacteria in Table 2 suggest differential regulation of these genes that may function simultaneously in other signal transduction pathways.

Activation of T and B lymphocytes to produce cytokines is linked to activation of programmed cell death responses in these cells. In this study, expression of mRNA for genes involved in apoptosis was reduced by the presence of probiotic bacteria (Table 2). Multiple signaling pathways of apoptosis induction and suppression function simultaneously to regulate this important process. We observed that probiotic bacteria were associated with suppression of caspase recruitment domain protein 10 (*Card10*), tumor necrosis factor alpha (*Tnf*), and fas-associated death domain protein (*Fadd*) mRNA expression, which are involved in initial steps of the apoptosis activation cascade and also reduction of mRNA expression of *Casp2* and *Casp12*, which are involved in the activation of enzymes involved in apoptosis (Table 2). Some genes involved in suppression of apoptosis cascade activation were also suppressed, including: defender against cell death 1 (*Dad1*), p21-activated kinase 7 (*Pak7*), v-akt murine thymoma viral oncogene homolog 1 (*Akt1*), *Bcl10*, and bcl2-associated agonist of cell death (*Bad*) (Table 2). These seemingly contradictory findings illustrate the precise balance needed in feedback regulation mechanisms for apoptosis in antigen-activated lymphocytes.

### Probiotic bacteria changed the tissue distribution of cellular activation markers

In addition to mRNA expression effects of probiotic bacteria, tissue distributions of several proteins were investigated. Immunohistochemical staining of MALT and mesenteric lymph node tissues was used to trace the tissue locations of cells with changes in production of CD21, CD2, PTPRC, RELB, and TLR6 proteins within the MALT tissues. Immunolocalization helped to interpret the qPCR results in terms of probiotic effects on host responses to *S. enterica* and these results will be presented in the following narrative that includes the observations and discussion of their significance in the context of T cells and antigen-presenting cells responding to *S. enterica* in the MALT and movement of these cells between MALT and MLN.

**Fig. 2:**
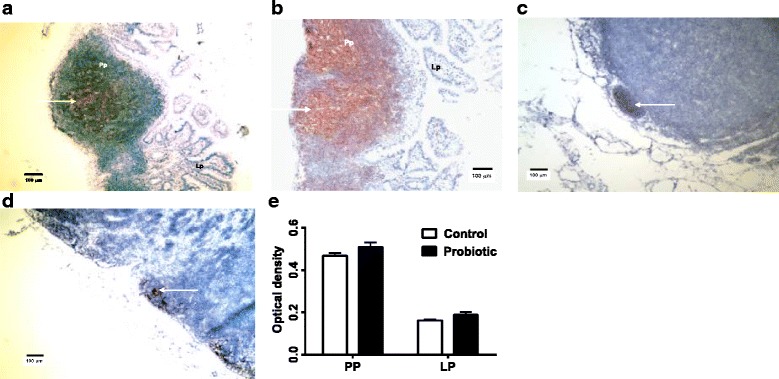
Immunohistochemical staining of CD21in Peyer’s Patches and MLN. The germinal centers of Peyer’s patches (Pp) (**a**, control mice; **b**, probiotic-treated mice) and MLN (**c**, control mice; **d**, probiotic-treated mice) contained numerous cells with CD21 (*arrows*) that have lymphocyte morphology. Images 10× magnification. There were no significant differences in optical density of stained cells in probiotic-treated Peyer’s patches (Pp) and lamina propria (Lp) compared to control *S. enterica*-challenged Peyer’s patches and lamina propria by ANOVA, *P* < 0.05 (**e**)

The CD21 protein is expressed on B lymphocytes and dendritic cells, which can be differentiated morphologically in MALT and MLN. The germinal centers of Peyer’s patches (Fig. 2a, b) and secondary follicles of MLN (Fig. 2c, d) contained numerous cells with CD21 that have lymphocyte morphology. Densitometry of the Peyer’s patches stained for CD21 suggested no change in the numbers of cells expressing this marker in the probiotic-treated group, (*P* = 0.055) by ANOVA (Fig. 2e). The increased CD21 mRNA expression in Peyer’s patches of the mice with probiotic treatment (Table 2) did not appear to change the numbers of CD21-producing cells in the Peyer’s patches.

**Fig. 3:**
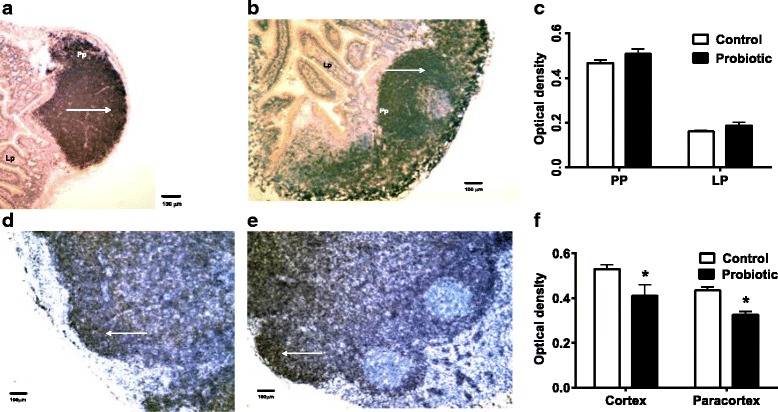
Immunohistochemical staining of CD2 in Peyer’s Patches and MLN. Peyer’s Patches (Pp) of *S. enterica*-infected mice were highly reactive with heavy follicular CD2- staining (arrows), especially in probiotic-treated mice (**a**, control mice; **b**, probiotic-treated mice). There were no significant differences in staining between control and treated Peyer’s patches or lamina propria (Lp) (**c**). CD2 staining of cortical and paracortical lymph node tissue (**d**, control mice; **e**, probiotic-treated mice) in probiotic-treated *Salmonella*-infected mice were different than control mice. Images 10× magnification. Statistically significant differences in staining of cortical and paracortical regions of the lymph nodes between control and probiotic-treated mice are shown at the asterisk, *P* < 0.05 (**f**)

Peyer’s patches of *S. enterica*-infected mice were highly reactive with heavy follicular CD2- staining (Fig. 3a). This is indicative of a strong migration of activated T cells to the site. There was reduced diffuse CD2 staining of cortical and paracortical lymph node tissue (Fig. 3e) in probiotic-treated *S. enterica*-infected mice, which was statistically significant between cortical and paracortical regions of the lymph nodes (Fig. 3f). These results are consistent with reduced inflammatory response in the probiotic-treated mice.

**Fig. 4:**
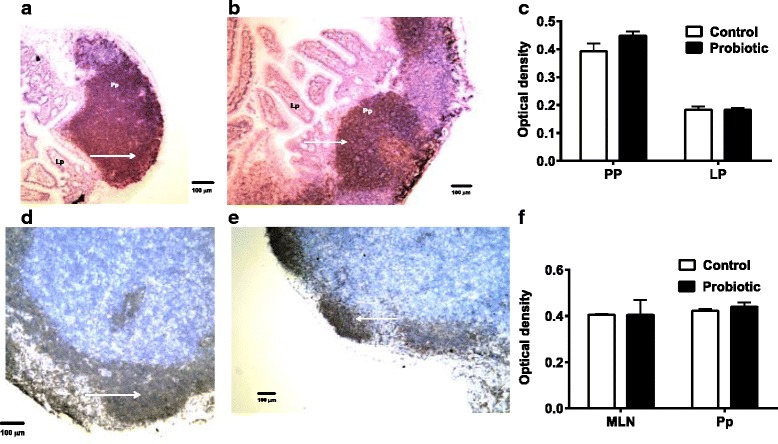
PTPRC production shown by immunohistochemistry. Peyer’s patches (Pp) and lamina propria (Lp) (**a**, control mice; **b**, probiotic-treated mice) appeared to have the same amount of PTPRC staining (arrows). There were no significant differences measured by densitometry (**c**). MLN (**d**, control mice; **e**, probiotic-treated mice) of probiotic-treated and *S. enterica*-infected mice also showed similar amounts of PTPRC staining in germinal centers. No significant differences in PTPRC staining between control and treated mice were confirmed by densitometry (**f**). PTPRC staining is shown at the arrows

The infiltration of PTPRC-expressing cells in Peyer’s Patches (Fig. 4a, b) and MLN (Fig. 4d, e) of probiotic treated and control *S. enterica*-infected mice in the two tissues appeared similar. Comparisons of staining densities of the replicates in these tissues of both treatment groups also showed no significant difference (Fig. 4c, f).

**Fig. 5:**
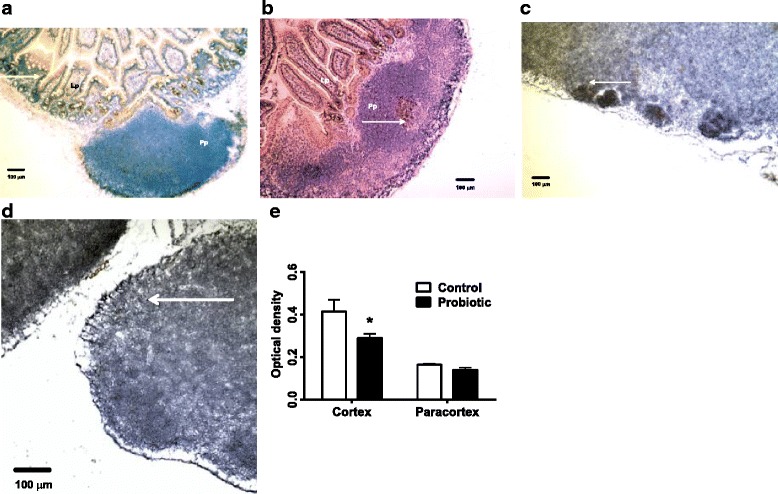
Immunohistochemical staining of TLR6. Cells were stained for TLR6 (*arrows*) in Peyer’s Patches (**a**, control mice; **b**, probiotic-treated mice) and MLN (**c**, control mice; **d**, probiotic-treated mice) from *S. enterica*-infected and probiotic-treated *S. enterica*-infected mice. The amount of TLR6 in cortical areas of probiotic-treated MLN was significantly reduced, *P* < 0.05 by ANOVA, compared with untreated *S. enterica*-challenged mice (**e**)

Expression of TLR6 was investigated immunohistochemically because its mRNA expression was increased in the probiotic-treated mice. TLR6 was not strongly stained in Peyer’s patches from probiotic-treated *S. enterica*-infected mice (Fig. 5b). The amount of TLR6 in cortical areas of probiotic-treated MLN (Fig. 5d), was significantly reduced, compared with untreated *S. enterica*-challenged mice (Fig. 5e).

**Fig. 6:**
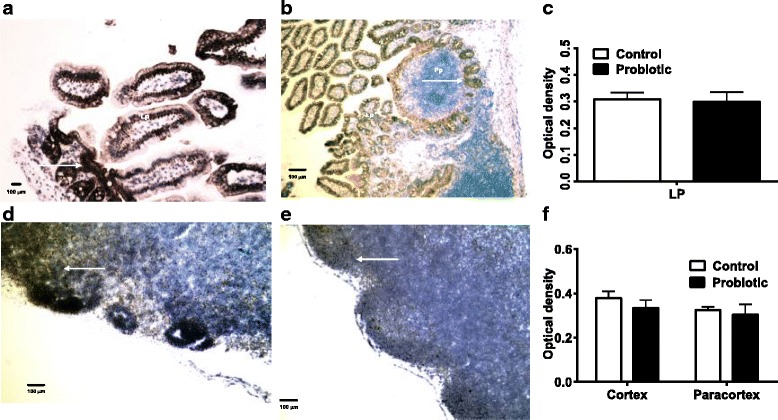
Immunohistochemical staining of RELB. The Peyer’s patches and lamina propria (**a**, control mice; **b**, probiotic-treated mice) and MLN of probiotic-treated *S. enterica*-infected mice (**d**, control mice; **e**, probiotic-treated mice) had RELB expressed in cells (*arrows*) with lymphocyte and epithelial cell morphologies. There were no significant differences in staining of lamina propria (Lp) of control or probiotic-treated mice (**c**). Staining in MLN from the probiotic-treated mice was not significantly different from controls, *P* < 0.05 by ANOVA (**f**)

RELB cortical staining was apparent in Peyer’s patches of probiotic-treated *S. enterica*-infected mice (Fig. 6b). Staining was also seen in MLN from the probiotic-treated mice (Fig. 6e) but was not significantly different between regions of the tissues or between treatments (Fig. 6c, f).

## Discussion

The concept of T cell depletion by early induction of a strong inflammatory response by *S. enterica* has been previously described [2, 3]. In the present study, we observed recovery of T cell proliferative responses in splenocytes from mice treated with the probiotic bacterial blend prior to *S. enterica* infection, which is consistent with the concept that T cell depletion was averted. In a previous report, an immunosuppressive effect of *S. enterica* infection was observed on splenic lymphoproliferative responses in gnotobiotic BALB/c mice [5]. Treatment of the mice with probiotic bacteria prevented the immunosuppressive effects. There are several immunosuppressive mechanisms used by virulent *S. enterica* to evade host responses, including elimination of high-avidity antigen-specific T cells [2], activation of suppressive regulatory T cells [3], and phagolysosomal escape in macrophages and dendritic cells [1, 7].

In the present study, the probiotic bacteria suppressed basal and LPS-induced splenic lymphoproliferation. A growing body of literature is reporting this characteristic of probiotic lactobacilli with inhibition of LPS-stimulated lymphocyte proliferation [8], inhibition of Concanavalin-A-stimulated proliferation [9], and anti-CD3ε-stimulated splenocyte proliferation [10]. It is consistent with the concept that pathogen-induced lymphocyte clonal deletion can be blocked by bacterial induction of mechanisms that inhibit proliferation. Cell surface receptors for commensal or probiotic bacteria may activate pathways that prevent proliferation and apoptosis of lymphocytes.

Bacterial activation of a pathway that inhibits lymphocyte activation and apoptotic caspase activity most likely occurs through the recognition of bacterial surface glycoproteins by TLR1, TLR2, or TLR6 on APC [11]. TLR4 (detects lipopolysaccharides-LPS) and TLR5 (detects flagellin) are also involved in the T cell activation response to *S. enterica* by APC [11]. In our present study, gene expression profiling qRT-PCR panels and immunohistochemistry were used to observe changes in expression of genes associated with lymphocyte activation and apoptosis and signal transduction from Toll-like receptors through the NF-κB and MAPK signal transduction pathways that showed evidence for a T cell-sparing effect from the influence of probiotic bacteria. Activation and signaling responses by lymphocytes are regulated by the NF-κB transcription factor and signal transduction pathway [12]. Some of the intracellular molecules that activate NF-κB include: CFLAR [13], CASP8 [13], conserved helix-loop-helix ubiquitous kinase (CHUK/IκκA) [14], RIPK1 [15], TRAF2 [15], and BCL10 [16].

Here we show evidence for a probiotic mechanism that prevents *S. enterica* immune evasion by T cell deletion through a mechanism of reduced inflammatory response early during the infection in BALB/c mice. The probiotic bacteria modulated expression of genes related to inflammation and T cell apoptosis, including some of the Toll-like receptors involved in *S. enterica*-induced inflammation. In this present study, we observed increased *Tlr6* mRNA in MALT of probiotic-treated mice compared with *S. enterica-*treated mice. This agrees with evidence that probiotic *Lactobacillus plantarum* induced *Tlr6* mRNA expression in intestinal epithelial cells of cyclophosphamide-suppressed mice [17]. We also observed that there was not an increase in TLR6 expressing cells in Peyer’s patches, suggesting a role for post-transcriptional regulation of TLR6 on the surface of lymphocytes in our mice. It is possible that TLR2 and TLR6 heterodimer activation can be inhibited by probiotics through activation of peptide regulators, as shown with TLR6 transmembrane domain peptides in vitro [18]. The latter paper suggested this inhibition is specific to TLR2, but the work was in vitro and may not show how the process may affect TLR2/TLR6 heterodimer surface expression in cells in vivo. Another possibility for this in the present study is that the different microbiota used in the human microbiota-associated mice versus the conventional mice may not have supported the probiotic effect.

Since we saw probiotic bacteria could reverse the suppression of splenic lymphocyte proliferation to *S. enterica* antigens, we looked at changes in expression of genes associated with T cell activation in mice treated with probiotic bacteria. Initial contacts by intestinal epithelial cells or professional phagocytes with *S. enterica* surface molecules that have pathogen-associated molecular patterns activate receptors that initiate signal transduction within the cell. These signals initiate mRNA transcription and protein synthesis of cytokines that are secreted into the extracellular environment and recruit inflammatory cells. When dendritic cells encounter *S. enterica* antigens in the intestinal tissues, they process the antigens for presentation to lymphocytes and migrate to MLN [19]. In the MLN, the dendritic cells activate antigen-specific T cells that are selected by stromal cells for expression of α4β7 integrin and CCR9, which is essential for their migration along a gradient of CCL25 and other chemokines to the intestinal MALT [20–22].

Clues to the probiotic mechanism that blocks *S. enterica*-induced immunosuppression might be seen in changes of expression of genes involved in recruitment of immune responses to the MALT and MLN of mice protected by the bacteria from *S. enterica* infection. MLN are essential for resistance to *S. enterica* infections, but are also the sites of long term persistence of infections [1]. The MLN hold *S. enterica*-infected dendritic cells and limit the dissemination of the infection by arresting DC migration [23]. Still, some of the bacteria can escape the MLN because host cell death can be induced by *S. enterica*, especially Serovar Typhimurium [1].

Some probiotic mechanisms do not involve immune and inflammatory cell migration. For example, cell-free spent media from probiotic *Bifidobacterium bifidum* inhibits growth of *S. enterica* [24]. These authors suggest that *Bifidobacter*-derived factors interfere with expression of *S. enterica* virulence genes encoded on the *Salmonella* pathogenicity islands 1 and 2. Some effects of commensal and probiotic bacteria appear to occur at the mucosal surface in epithelial cells. Some surface layer proteins of *Lactobacillus acidophilus* inhibit CASP3 responses to *Salmonella* sp. in IEC cell lines [25] thus, reducing apoptotic death and loss of epithelial barrier functions to *Salmonella* sp. invasion. Clearly, evidence exists for the combination of microbial interactions for colonization resistance to *S. enterica* infections and influences on the innate and adaptive immune responses can be attributed to probiotic bacteria.

## Conclusions

Despite all the efforts of public health and agricultural programs to reduce acquisition of *Salmonella* sp. infections, they persist as a major health issue. One reason for this is the asymptomatic carrier state of some individuals who have recovered from acute infections. Persistence of a carrier state is facilitated by immune system evasion mechanisms inherent to the genus. Strains of bacteria in the intestinal microbiota and found in probiotic products can antagonize *Salmonella* sp. infections, perhaps by inhibition of these inherent immune system evasion mechanisms. In this study, the presence of probiotic bacteria in the intestinal tracts of mice during *S. enterica* infections prevented loss of splenocyte proliferation and decreased splenocyte apoptosis. Gene expression data showed changes that suggest lymphocyte activation, survival. And immune cell homing functions were restored, This was related to an apparent ability of the probiotic bacteria to limit exhaustive lymphocyte proliferation and apoptosis. The migration of lymphocytes and dendritic cells in intestinal mucosal tissues was restored by the presence of the probiotic bacteria. These results are consistent with the theory that *S. enterica* induced clonal deletion of lymphocytes that was inhibited by the presence of probiotic bacteria.

## Abbreviations

AKT1: v-akt murine thymoma viral oncogene homolog 1
APC: Antigen presenting cell
BAD: bcl2-associated agonist of cell death
BCL2: B- cell CLL/lymphoma 2
CARD10: Caspase recruitment domain protein 10
CASP2: Apoptosis-related cysteine peptidase 2
CD2: Clusters of differentiation antigen 2
CFLAR: CASP8 and FADD like apoptosis regulator
CFU: Colony forming unit
DAD1: Defender against cell death 1
FADD: Fas-associated death domain protein
IL4: Interleukin-4
IRAK2: Interleukin-1 receptor-associated kinase 2
IκκB: Inhibitor of light polypeptide gene enhancer in B cell kinase alpha
JUN: v-jun sarcoma virus 17 oncogene homolog
LPS: Lipopolysaccharide
MALT: Mucosa associated lymphoid tissue
MEKK: Map/Erk kinase kinase
MLN: Mesenteric lymph node
MRS: de Man Rogosa Sharpe
MTS: 3-(4,5-dimethylthiazol-2-yl)-5-(3-carboxymethoxyphenyl)-2-(4-sulfonyl)-2H–tetrazolium, inner salt
MYD88: Myeloid differentiation primary response gene 88
NF-κB: Nuclear factor of kappa light chains enhancer in B cells
PAK7: p21-activated kinase 7
PTPRC: Protein tyrosine phosphatase receptor type C
qRT-PCR: quantitative real-time polymerase chain reaction
RELB: v-Rel avian reticuloendotheliosis viral oncogene homolog B
RIPK1: Receptor TNFRSF-interacting serine threonine kinase 1
RNF7: Ring finger protein 7
RPMI 1640: Roswell Park Memorial Institute 1640
SEM: Standard error of the mean
TLR6: Toll-like receptor 6
TNF: Tumor necrosis factor alpha
TRAF2: TNF receptor associated factor 2
